# Trends in Unintentional Injury Mortality Among Children and Adolescents in Poland, 2010–2024: An Unfinished Lesson for Public Health in Changing Social Realities

**DOI:** 10.2478/sjph-2026-0018

**Published:** 2026-09-01

**Authors:** Rafał Halik, Mariusz Duplaga

**Affiliations:** National Institute of Public Health NIH - National Research Institute, Department of Population Health Monitoring and Analysis, ul. Chocimska 24, 00-791 Warszawa, Poland; Jagiellonian University Medical College, Institute of Public Health, Department of Health Promotion and e-Health, ul. Skawińska 8, 31-066 Kraków, Poland

**Keywords:** Unintentional injuries, Child mortality, Poland, Time trends, Injury prevention, Rural–urban disparities, nezgode, umrljivost otrok in mladostnikov, Poljska, časovni trendi, preprečevanje nezgod, razlike med mestom in podeželjem

## Abstract

**Introduction:**

Unintentional injuries are a major cause of death among children and adolescents in Europe. Poland has historically reported elevated mortality levels. This study assesses national trends in injury mortality among individuals aged 1–19 years from 2010 to 2024.

**Methods:**

A national time-trend analysis was conducted using Statistics Poland death registry data. Mortality rates were calculated by age group, sex, residence, and cause of death. Trends were modelled using log-linear Poisson regression to estimate annual percent change (APC). Global Burden of Disease estimates provided international context.

**Results:**

A total of 6,812 deaths from unintentional injuries occurred during 2010–2024. Overall mortality decreased from 8.5 to 4.9 per 100,000 (APC −4.8%; 95% CI −5.3 to −4.3). The highest average mortality rate during 2010–2024 was observed in the 15–19 age group (15.4; 95% CI 14.9–15.8). Rural areas showed the steepest improvement (APC −6.7%), with rates declining from 11.7 in 2010 to 4.7 in 2024. The fastest age-specific decline occurred among children aged 5–9 years (APC −6.7%). Transport-related injuries contributed approximately 71% of the total decline. Drowning mortality showed the most pronounced relative decrease (APC −10.0%), while poisoning mortality increased significantly (APC +6.3%).

**Conclusions:**

Mortality from unintentional injuries among children and adolescents in Poland has declined to levels comparable with the European Union; however, these improvements have been uneven across population groups. Further progress will require adapting prevention strategies to increasingly complex and evolving risk factors shaped by changing societal conditions.

## Izvleček

**Uvod:**

Nezgode so eden glavnih vzrokov smrti med otroki in mladostniki v Evropi. Poljska že dlje časa beleži višje stopnje umrljivosti zaradi nezgod. Ta študija ocenjuje nacionalne trende umrljivosti zaradi nezgod med osebami, starimi od 1 do 19 let v obdobju od leta 2010 do leta 2024.

**Metode:**

Na podlagi podatkov iz registra smrti Poljske statistične službe je bila izvedena nacionalna analiza časovnih trendov. Stopnje umrljivosti so bile izračunane po starostnih skupinah, spolu, kraju bivanja in vzroku smrti. Trendi so bili modelirani z uporabo log-linearne Poissonove regresije za oceno letne odstotne spremembe (APC). Ocene globalnega bremena bolezni so zagotovile mednarodni kontekst.

**Rezultati:**

V obdobju 2010–2024 je zaradi nezgod umrlo skupno 6.812 oseb. Splošna umrljivost se je zmanjšala z 8,5 na 4,9 na 100.000 prebivalcev (APC −4,8 %; 95-% IZ: od −5,3 do −4,3). Najvišja povprečna stopnja umrljivosti v obdobju 2010–2024 je bila ugotovljena v starostni skupini 15–19 let (15,4; 95-% IZ: 14,9–15,8). Do največjega izboljšanja je prišlo na podeželju (APC −6,7 %), pri čemer so se stopnje umrljivosti zmanjšale z 11,7 leta 2010 na 4,7 leta 2024. Najhitrejši upad umrljivosti glede na starost je bil zabeležen pri otrocih, starih od 5 do 9 let (APC−6,7 %). K skupnemu zmanjšanju umrljivosti so poškodbe v prometu prispevale približno 71 %. Umrljivost zaradi utopitev je pokazala najbolj relativni upad (APC −10,0 %), medtem ko se je umrljivost zaradi zastrupitev znatno povečala (APC +6,3 %).

**Zaključki:**

Umrljivost zaradi nezgod med otroki in mladostniki na Poljskem se je zmanjšala na raven, primerljivo z ravnjo v Evropski uniji, vendar napredek ni bil enakomeren med vsemi skupinami prebivalstva. Nadaljnji napredek bo zahteval prilagajanje preventivnih strategij vse bolj kompleksnim in spreminjajočim se dejavnikom tveganja, ki jih oblikujejo družbene spremembe.

## 1 INTRODUCTION

Accidents (unintentional injuries) represent a major health burden for children, accounting for 17% of deaths among children and adolescents and 10% of years of life lost (DALY) due to unintentional injuries across the entire European Union; the burden is particularly high in adolescents (32% of deaths and 12% of DALYs) (1).

Alongside substantial reductions in neonatal mortality and advances in the prevention and treatment of childhood diseases, declines in injury mortality have been among the most significant drivers of reductions in child mortality in Europe (2, 3). Mortality analyses conducted prior to the COVID-19 pandemic suggest that, although Poland's mortality rates remained above the EU average, they declined more steeply than the EU average (4, 5). Nevertheless, the situation in Europe remains challenging. Despite being largely preventable, accidents among children and adolescents continue to rank among the leading health threats to this population, and progress has unfolded mostly through fragmented, sector-specific initiatives such as road safety strategies, technical standards, regulatory measures, and education rather than through an overarching, system-oriented, and wellcoordinated child injury prevention framework (6, 7).

In Poland, the burden of unintentional injury mortality among children has historically been elevated in farming areas, where children often face dual exposure: routine participation in family farm tasks and involvement in higher-severity road crashes. Representative studies document substantial adolescent involvement in farm work (70%) and a non-negligible injury history (18%), with typical mechanisms including falls, slips, and contact with machinery in and around farm facilities, meadows, and fields (8, 9). Similarly, on rural roads, vulnerable road users, particularly pedestrians, have experienced disproportionate case-fatality rates, with alcohol involvement, rural location, and environmental factors such as darkness and unsignalized crossings identified as key risk amplifiers (10).

This study examines the magnitude and temporal trends of unintentional injury mortality among Polish children and adolescents aged 1–19 years during 2010–2024, with a particular focus on the leading external causes. The objectives are to: (i) characterise demographic patterns; (ii) compare rural and urban settings. The findings are intended to identify priority domains for health promotion and further research.

## 2 METHODS

### 2.1 Data sources

Mortality data for Poland were obtained from the national death registry maintained by Statistics Poland (GUS) (11). The underlying cause of death was coded according to the International Classification of Diseases, 10th Revision (ICD-10) and used to identify unintentional external causes and to classify deaths into specific injury mechanisms.

The following ICD-10 codes were used for the classification of unintentional injuries:

1. Accidents (V01–X59)
2. Accidents with pedestrians (V01–V09)
3. Car-occupant road-traffic injuries (V40–V49)
4. Other transport injuries (V10–V39 and V50–V99)
5. Drowning (W65–W74)
6. Falls (W00–W19)
7. Burns and fire exposure (X00–X09)
8. Poisonings (X40–X49)
9. Other unintentional injuries (residual category) all remaining external cause codes

Annual population denominators by age group (1–4, 5–9, 10–14, 15–19 years), sex, and place of residence (urban/rural) were obtained from GUS (11).

To enable international comparisons, data from the Global Burden of Disease (GBD) study were used for Poland, neighbouring countries (Germany, Czechia, Slovakia, Ukraine, Belarus, Lithuania, and Russia), and the European Union (EU). GBD estimates were applied consistently across all locations, including Poland, to ensure comparability. The most recent GBD release available at the time of analysis was used, providing annually resolved, model-based mortality estimates (12).

### 2.2 Statistical analysis

Mortality rates were calculated per 100,000 population with 95% confidence intervals (CIs), assuming a Poisson distribution of counts. Age standardisation was not performed because the analysis was conducted within narrow, fixed 5-year age groups; in this context, direct standardisation may introduce instability when estimates are stratified by sex, residence, and cause. Accordingly, crude and period-average rates with 95% CIs are presented for each stratum and overall.

Temporal trends were modelled using log-linear Poisson regression with the logarithm of the population as an offset (13) to estimate annual percent change (APC) with 95% CIs and p-values. APC estimates are presented for Poland overall and stratified by age × sex, residence × sex, and cause × residence.

All statistical analyses, including APC estimation, were performed in R using generalised linear models with a Poisson distribution.

## 3 RESULTS

### 3.1 Mortality rates

**Figure 1:**
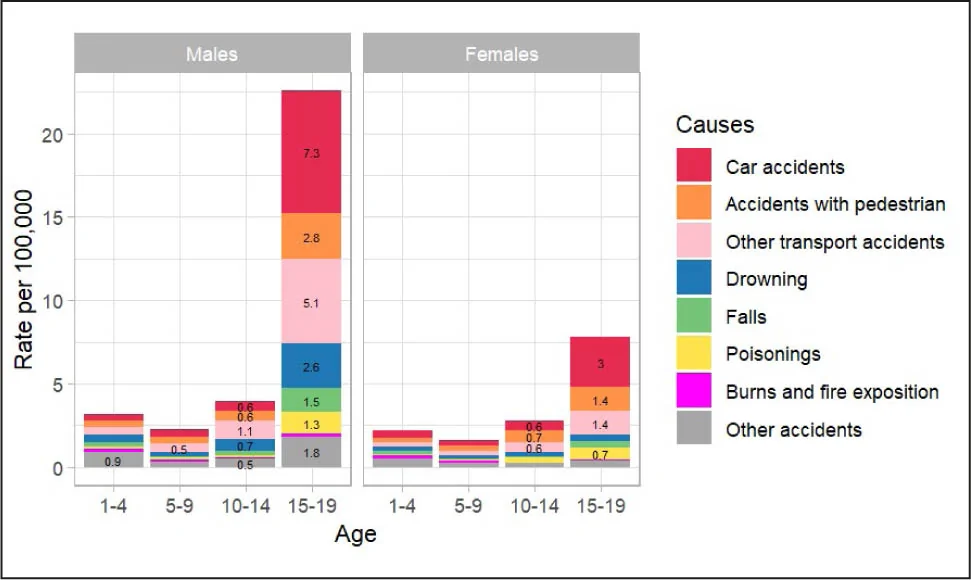
Period-average mortality rates (2010–2024) by external causes of unintentional injury.

During the analysed period (2010–2024), a total of 6,812 deaths from unintentional injuries were recorded among children and adolescents aged 1–19 years. Males accounted for more than 70% of all deaths. Average mortality rates over the period 2010–2024 exhibited a U-shaped pattern across age groups (1–4, 5–9, 10–14, and 15–19 years), with the lowest mortality observed at ages 5–9 years, slightly higher rates in early childhood (1–4 years), and a marked increase from ages 10–14 years onward, peaking sharply in the 15–19-year age group. In younger children, mortality was generally low, particularly in the 5–9-year group, where no single accident category exceeded 1 per 100,000. The average rates for the years 2010–2024 were 2.7 (95% CI: 2.5–2.9) at ages 1–4, 2.0 (1.8–2.1) at 5–9, 3.4 (3.2–3.6) at 10–14, and 15.4 (14.9–15.8) at 15–19. At all ages, mortality was higher among males than females (Figure 1).

Transport-related injuries were the leading cause of death across all age groups and in both sexes based on average rates for 2010–2024. Among males aged 15–19 years, the highest mortality rates were observed for car-occupant injuries (approximately 7.3 per 100,000), followed by other transport injuries (5.1 per 100,000) and pedestrian injuries (2.8 per 100,000). Drowning (2.6 per 100,000) and falls (1.5 per 100,000) were less frequent but remained notable causes. A similar pattern was observed among females aged 15–19 years, although at lower levels, with the highest rates for car-occupant injuries (3.0 per 100,000) and pedestrian injuries (1.4 per 100,000).

Unintentional injury mortality was consistently higher in rural than in urban areas for both sexes (Table 1). The highest unintentional injury mortality was observed among rural males aged 15–19 years (28.5, 95% CI: 27.3–29.8). Among boys, rural rates exceeded urban rates at all ages, with particularly large differences at ages 15–19 and clear rural disadvantages already evident at ages 1–4 and 10–14 (non-overlapping confidence intervals). Among girls, rural rates were also higher across all age groups, with a clear difference at ages 15–19 (8.8 [8.1–9.5] vs 7.0 [6.5–7.6]); at younger ages, rural–urban differences were smaller and confidence intervals partially overlapped.

**Table 1:** Average crude death rates per 100,000 due to unintentional injuries for years 2010–2024 in Poland by gender, age group, and place of residence.

**Sex**	**Age group**	**Urban areas – rate and 95% CI**	**Rural areas – rate and 95% CI**	**Poland-rate and 95% CI**
**Overall**	**Overall**	**4.9 (4.7–5.1)**	**7.6 (7.3–7.8)**	**6.1 (5.9–6.2)**
Overall	1–4	2.2 (2–2.5)	3.4 (3.0–3.8)	2.7 (2.5–2.9)
Overall	5–9	1.7 (1.5–1.9)	2.3 (2–2.6)	2.0 (1.8–2.1)
Overall	10–14	2.9 (2.6–3.1)	4.0 (3.7–4.3)	3.4 (3.2–3.6)
Overall	15–19	12.3 (11.8–12.9)	18.9 (18.2–19.7)	15.4 (14.9–15.8)
**Males**	**Overall**	**6.4 (6.1–6.7)**	**10.7 (10.3–11.1)**	**8.3 (8.1–8.6)**
Males	1–4	2.5 (2.1–2.9)	4.2 (3.6–4.8)	3.2 (2.9–3.5)
Males	5–9	2.0 (1.7–2.3)	2.6 (2.3–3.1)	2.3 (2–2.5)
Males	10–14	3.1 (2.7–3.5)	5.0 (4.5–5.5)	3.9 (3.6–4.3)
Males	15–19	17.4 (16.5–18.3)	28.5 (27.3–29.8)	22.5 (21.8–23.3)
**Females**	**Overall**	**3.3 (3.1–3.6)**	**4.2 (4.0–4.5)**	**3.7 (3.6–3.9)**
Females	1–4	2.0 (1.6–2.3)	2.5 (2.1–3.0)	2.2 (1.9–2.5)
Females	5–9	1.5 (1.2–1.7)	1.9 (1.6–2.3)	1.7 (1.5–1.9)
Females	10–14	2.7 (2.3–3.1)	2.9 (2.5–3.4)	2.8 (2.5–3.1)
Females	15–19	7.0 (6.5–7.6)	8.8 (8.1–9.5)	1.8 (7.4–8.3)

### 3.2 Trends of mortality

**Figure 2:**
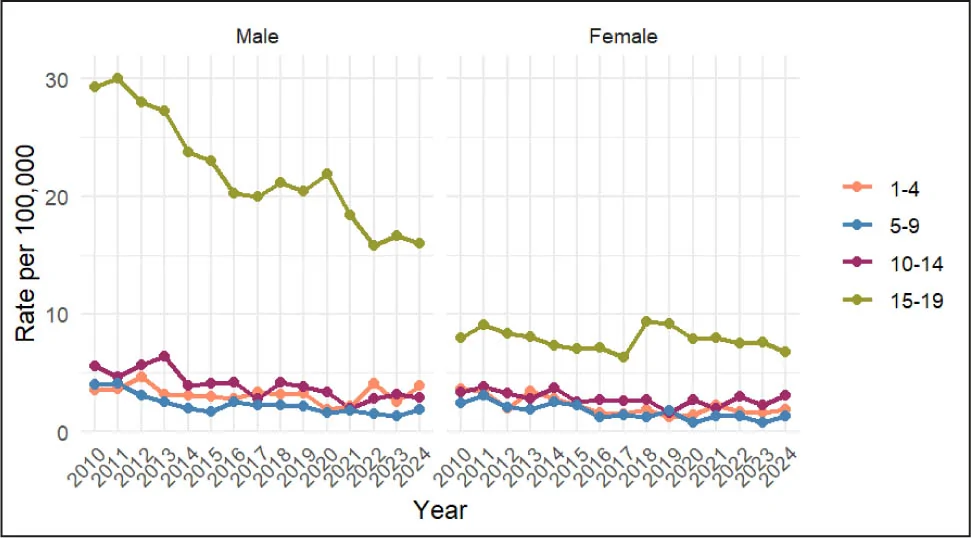
Crude death rates due to unintentional injuries among children and adolescents in Poland (2010–2024) by age and sex.

Stratification by age and sex revealed heterogeneous temporal patterns across demographic subgroups (Figure 2, Table 2). The most substantial declines in mortality were observed among school-age children, with the 5–9 years age group demonstrating the steepest reduction (APC −6.7%; 95% CI: −8.5 to −4.5), followed by those aged 10–14 years (APC −4.3%; 95% CI: −5.7 to −2.9) and 15–19 years (APC −3.5%; 95% CI: −4.1 to −2.9). The youngest age group (1–4 years) also showed a statistically significant downward trend, similar to adolescents (APC −3.4; 95% CI: −5.1 to −1.6). When examined by sex, males experienced a markedly steeper decline in mortality than females. Male mortality decreased from 12.2 to 6.3 per 100,000 (APC −5.6; 95% CI: −6.2 to −4.9), nearly twice the rate observed in females, whose mortality declined from 4.6 to 3.4 per 100,000 (APC −3.0; 95% CI: −4.0 to −2.0). This differential trajectory suggests that the larger absolute reductions occurred mainly in the group with the higher baseline risk. Within gender-specific subgroups, the most pronounced and consistent improvements were concentrated among school-age boys and girls. Males aged 5–9 years showed an APC of −6.5% (95% CI: −8.9 to −4.1), while those aged 10–14 years had an APC of −5.4% (95% CI: −7.2 to −3.6). Similarly, females aged 5–9 years demonstrated the sharpest decline of female subgroups (APC −6.8%; 95% CI: −9.6 to −3.8). Notably, two subgroups did not demonstrate statistically significant trends: males aged 1–4 years (APC −1.7%; 95% CI: −4.4 to 0.6) and females aged 15–19 years (APC −0.6%; 95% CI: −1.9 to 0.7).

**Table 2:** Trends of death rates per 100,000 due to unintentional injuries by age and gender in Poland (2010 vs 2024) with Average Percentage Change (APC).

**Gender**	**Age group**	**APC (%) and 95% CI**	**Rate in 2010**	**Rate in 2024**
**Overall**	**Overall**	**−4.8 (−5.3; −4.3)**	**8.5**	**4.9**
Overall	1–4	−3.4 (−5.1; −1.6)	3.6	2.9
Overall	5–9	−6.7 (−8.5; −4.8)	3.2	1.6
Overall	10–14	−4.3 (−5.7; −2.9)	4.5	3.0
Overall	15–19	−3.5 (−4.1; −2.9)	18.8	11.5
**Males**	**Overall**	**−5.6 (−6.2; −4.9)**	**12.2**	**6.3**
Males	1–4	−1.7 (−4; 0.6)	3.5	3.9
Males	5–9	−6.5 (−8.9; −4.1)	4.0	1.9
Males	10–14	−5.4 (−7.2; −3.6)	5.5	2.9
Males	15–19	−4.5 (−5.2; −3.7)	29.3	16.0
**Females**	**Overall**	**−3.0 (−4.0; −2.0)**	**4.6**	**3.4**
Females	1–4	−5.9 (−8.7; −3.1)	3.7	1.9
Females	5–9	−6.8 (−9.6; −3.9)	2.4	1.4
Females	10–14	−2.6 (−4.8; −0.4)	3.4	3.0
Females	15–19	−0.6 (−1.9; 0.7)	7.9	6.7

Analyses of children and adolescents residing in rural and urban areas, stratified by sex, revealed distinct temporal trajectories. The sharpest improvements occurred in rural settings, particularly among males (drop from 17.0 to 6.0 per 100,000; APC −7.3%; 95% CI: −8.2 to −6.5) and females (6.1 to 3.3; APC −5.1%; 95% CI: −6.4 to −3.7). A different pattern emerged in urban areas, where mortality among males declined more modestly (from 8.2 to 6.5 per 100,000; APC −3.1%; 95% CI: −4.50 to −1.21), while female mortality remained essentially stable (3.4 per 100,000; APC −0.9%; 95% CI: −2.3 to 0.5; not statistically significant). Consistent with these subgroup patterns, overall rural mortality fell steeply (11.7 to 4.7 per 100,000; APC −6.7%; 95% CI: −7.4 to −6.0), whereas overall urban mortality decreased only slightly (5.9 to 5.0 per 100,000; APC −2.4%; 95% CI: −3.2 to −1.6). By 2024, the rapid decline in rural areas, combined with only a modest decrease in urban settings, resulted in similar levels of unintentional injury mortality among children and adolescents across both settings.

**Figure 3:**
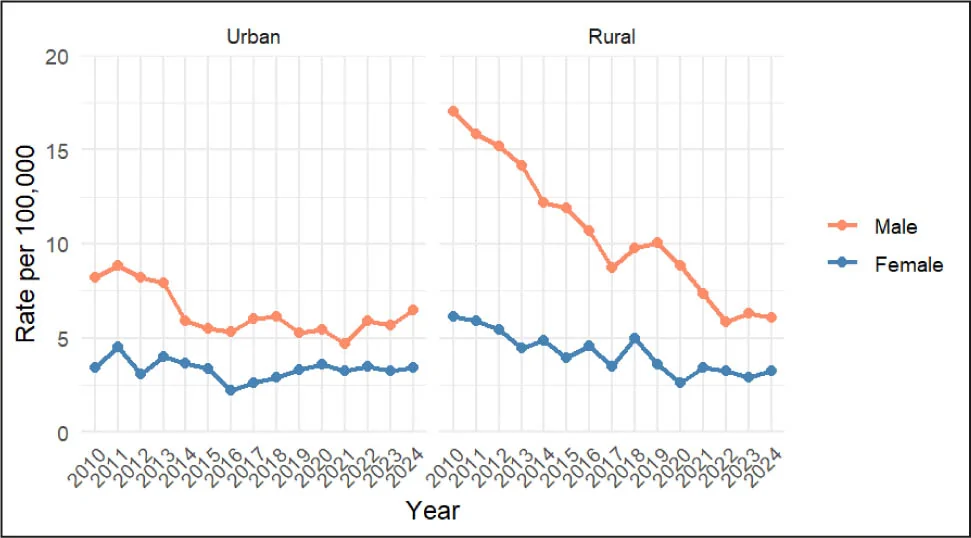
Crude death rates due to unintentional injuries among children and adolescents in Poland (2010–2024) by setting of residence.

**Table 3:** Trends of death rates per 100,000 due to unintentional injuries by gender in Poland (2010 vs 2024) with Average Percentage Change (APC).

**Gender**	**Setting of Residence**	**APC (%) and 95% CI**	**Rate in 2010**	**Rate in 2024**
**Overall**	**Urban**	**−2.4 (−3.2; −1.6)**	**5.9**	**5.0**
Males	Urban	−3.1 (−4.1; −2.2)	8.2	6.5
Females	Urban	−0.9 (−2.3; 0.5)	3.4	3.4
**Overall**	**Rural**	**−6.7 (−7.4; −6.0)**	**11.7**	**4.7**
Males	Rural	−7.3 (−8.2; −6.5)	17.0	6.0
Females	Rural	−5.1 (−6.4; −3.7)	6.1	3.3

Between 2010 and 2024, the overall accident mortality rate among children and adolescents fell from 8.5 to 4.9 per 100,000 (absolute decline: 3.6 per 100,000). The largest single contributor to this decline was car accidents (−1.62 per 100,000; approximately 45% of the total reduction), followed by drowning (−0.87; 24%), pedestrian transport injuries (−0.47; 13%), and other transport injuries (−0.46; 13%). Taken together, transport accidents: car, pedestrian, and other transport accidents accounted for 71% of the overall decline (combined −2.55 per 100,000 of −3.62). Smaller additional contributions came from other accidents (−0.34; 9%) and falls (−0.08; 2%), while poisonings increased (+0.22), partially offsetting gains (+6% of the net change). By sex, the decline among males (−5.87) was led by car-occupant transport injuries (−2.21; 38%) and drowning (−1.53; 26%), with additional contributions from other transport (−0.96) and pedestrian transport injuries (−0.67). Among females (decline −1.27 per 100,000), the principal driver was car-occupant transport injuries (−1.01; 80%), with smaller contributions from pedestrian transport injuries (−0.26) and drowning (−0.18)

Patterns by place of residence and sex were consistent with these category-level contributions. In rural areas (overall decline −7.02), the reduction was driven primarily by car-occupant transport injuries (−2.71; 39%), drowning (−1.44; 20%), and pedestrian transport injuries (−1.08; 15%). In urban areas (overall decline −0.85 per 100,000), the decrease was modest and concentrated in car-occupant transport injuries (−0.73; 86%) and drowning (−0.41), but partly offset by a rise in poisonings (+0.27).

All unintentional injury mortality declined (Table 4) at APC −4.8% (95% CI: −5.3; −4.3). The greatest improvement was observed in drowning (APC −10.0%; 95% CI: −11.7; −8.5), followed by car-occupant transport injuries (APC −5.9%; 95% CI: −6.9; −4.9) and accidents with pedestrians (APC - 4.90% 95%CI −6.3;−3.5). In contrast, poisonings increased over the period (APC +6.3%; 95% CI: 3.9 to 8.7), representing the only cause with a sustained upward trajectory and partially offsetting gains from the leading declining categories. Collectively, the uniformly favourable trends across transport-related causes (car, pedestrian, other transport) and the marked improvement in drowning are consistent with the principal drivers of the overall downward trend.

**Table 4:** Trends of death rates per 100,000 due to unintentional injuries by category of accidents in Poland (2010 vs 2024) with Average Percentage Change (APC).

**Category of accidents**	**APC (%) and 95%CI**	**Rate in 2010**	**Rate in 2024**
**All accidents**	**−4.8 (−5.3; −4.3)**	**8.5**	**4.9**
Car accidents	−5.9 (−6.9; −4.9)	2.6	1.0
Drowning	−10.1 (−11.7; −8.5)	1.3	0.4
Accidents with pedestrians	−4.9 (−6.3; −3.5)	1.2	0.7
Other transport accidents	−4.6 (−5.8; −3.5)	1.8	1.3
Falls	−3.9 (−6; −1.8)	0.3	0.2
Other accidents	−3.5 (−5; −2)	1.0	0.7
Poisonings	6.3 (3.9; 8.7)	0.3	0.5

Note: Burns and fire exposure were excluded as an independent category due to the low number of deaths.

### 3.3 Comparisons of mortality from Global Burden of Disease

**Figure 4:**
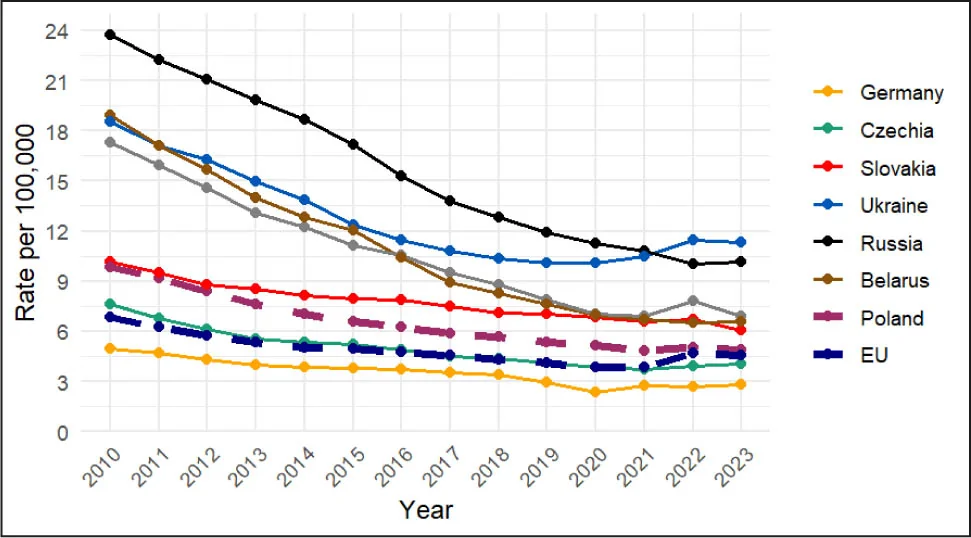
Unintentional injury mortality rates among children and adolescents (< 20 years), based on GBD estimates (2010–2023).

Using GBD estimates for 2010–2023, Poland experienced a pronounced decline in accident mortality among children and adolescents (Figure 4), decreasing from 9.9 to 4.9 deaths per 100,000, corresponding to an average annual percentage change (APC) of −5.4% (95% CI: −6.1; −4.8). This decline occurred at a faster rate than that observed for the EU average (APC −3.4%; 95% CI: −4.6; −2.2), leading to a substantial convergence of mortality levels by the late 2010s. By 2023, accident mortality in Poland and the EU average were essentially comparable (4.9 vs 4.6 deaths per 100,000, respectively). From approximately 2019 onwards, the downward trend in Poland flattened, with a pattern similar to that observed for the EU, where the trend stabilised and showed a slight increase in the most recent years. Compared with neighbouring countries, Poland's decline was comparable to trends in Germany (−5.0%) and Czechia (−5.0%). In contrast, Ukraine (−4.3%) and Slovakia (−3.4%) showed more moderate reductions. Overall, the Polish trajectory aligns with the broader regional pattern of declining accident mortality followed by stabilisation in the late 2010s, differing mainly in the earlier, more rapid rate of decrease relative to the EU average.

## 4 DISCUSSION

The findings indicate that Poland has achieved substantial progress in reducing mortality from unintentional injuries among children and adolescents, reaching levels broadly comparable to the European Union. This convergence suggests that the most effective prevention domains (14), particularly those related to transport safety, have largely matured, and that further gains may require a shift toward more targeted and context-specific health promotion strategies.

From a health promotion perspective, combating psychoactive substance use constitutes one of the important elements in injury prevention among adolescents. Reducing injury risk requires addressing both early initiation and ongoing use of such substances. According to the European ESPAD survey published in 2024 (15), alcohol remains the most prevalent substance among European adolescents (approximately 73% lifetime use), with Poland showing comparable levels (71% lifetime), though slightly lower in declarations of recent use (37% vs. 42% across participating countries). Another survey conducted in Poland, published in 2018 (16), reported limited parental awareness of adolescent alcohol use (approximately 49.5%). This study also showed that up to one-third of parents may accept underage drinking, indicating a permissive social environment that may facilitate the normalisation of such behaviours. Regarding illicit drugs, ESPAD data indicate that lifetime use in Poland (approximately 17%) is slightly higher than the European average (13%), suggesting broadly similar yet somewhat elevated exposure. An emerging concern in Poland is vaping, which has become highly prevalent among adolescents: according to ESPAD, up to 20% reported use in the past 30 days, compared to 8.8% across Europe, representing the highest levels observed among all participating countries. Importantly, e-cigarettes also serve as a route of psychoactive substance intake. According to a Polish cross-sectional study published in 2025 (17), 7.15% of adolescents reported adding substances such as THC to e-cigarette liquids, indicating an evolving risk environment. Taken together, these findings suggest that patterns of substance use among adolescents are changing in Poland. While alcohol has historically been the dominant concern in Poland and Central-Eastern Europe, the coexistence of high vaping prevalence, stable or slightly elevated illicit drug use, and permissive social norms represents a particularly concerning public health challenge.

The uneven distribution of improvement across population subgroups points to changing epidemiological dynamics. While substantial gains were observed in rural areas, the relative stabilisation of mortality in urban settings since approximately 2015 suggests that existing interventions may be less effective in environments where risk determinants are evolving. Adolescents are increasingly exposed to new behavioural drivers shaped by social media ecosystems and digital marketing, which influence risk perception, social norms, and engagement in potentially hazardous activities (18). From a health promotion perspective, this indicates the need to move beyond universal approaches toward interventions that better reflect local context, behavioural patterns, and social environments, particularly among adolescents in urban areas. In our study, the decline in unintentional injury mortality among girls was smaller than among boys. This suggests that injury prevention efforts may not equally benefit both sexes and that risk factors specific to girls remain insufficiently addressed. One explanation may involve differences between genders in accident types and the risk factors related to them.

The increasing importance of poisoning-related mortality highlights an area where preventive approaches need to be further differentiated by age. In younger children, poisoning risk is primarily linked to accidental exposure and is therefore likely to be preventable through improved parental awareness and reduced accessibility of toxic substances (19). In contrast, among adolescents, poisoning increasingly intersects with behaviours such as substance use and may also reflect intentional self-harm (20). These mechanisms are more complex and suggest the need for integrating injury prevention with mental health promotion, substance use prevention, and school-based interventions targeting risk behaviours. At the same time, the interpretation of accidental poisoning mortality remains constrained by limitations of cause-of-death data, including the potential misclassification between unintentional and intentional injuries (21, 22). This issue is particularly relevant in Poland, where previous studies have shown that deaths due to poisoning and events of undetermined intent may partially conceal suicides, indicating that some cases of self-harm are recorded as accidental deaths in official mortality statistics (23).

A similar need for conceptual expansion applies to transport-related injury prevention. While traditional road safety measures have contributed substantially to past improvements, emerging forms of mobility such as electric scooters and other micro-mobility devices are likely to be reshaping patterns of exposure, particularly in urban settings (24). These developments may introduce new risk environments that are not fully captured by existing prevention frameworks and require updated regulatory, infrastructural, and educational responses.

More broadly, effective health promotion in the area of children's injuries will depend on the ability to align epidemiological monitoring with adaptive intervention strategies. Recognising shifts in risk distribution and underlying determinants is essential to sustain progress and to prevent stagnation in reducing injury-related mortality.

## 5 CONCLUSIONS

Mortality from unintentional injuries among children and adolescents in Poland has declined to levels comparable with the European Union; however, these improvements have been uneven across population groups. Further progress will require adapting prevention strategies to increasingly complex and evolving risk factors shaped by changing societal conditions. Continued research and surveillance are essential to better understand these dynamics and support targeted, effective health promotion interventions.
